# PAX2 may induce ADAM10 expression in renal tubular epithelial cells and contribute to epithelial-to-mesenchymal transition

**DOI:** 10.1007/s11255-018-1956-0

**Published:** 2018-08-16

**Authors:** Ling Hou, Yue Du, Chengguang Zhao, Yubin Wu

**Affiliations:** Department of Pediatric Nephrology, Shengjing Hospital of China Medical University, Shenyang, 110004 Liaoning China

**Keywords:** ADAM10, Chronic kidney disease, Epithelial-to-mesenchymal transition, PAX2, Renal fibrosis, Renal tubular epithelia

## Abstract

**Purpose:**

We sought to investigate the role of PAX2 in renal epithelial-to-mesenchymal transition (EMT), examining the influence of PAX2 on ADAM10 expression during renal EMT and ADAM10 expression in fibrotic kidneys.

**Methods:**

A rat renal tubular epithelial cell line, NRK52E, was transfected with lentivirus carrying PAX2, and E-cadherin and α-SMA expressions were measured. The influence of PAX2 on ADAM10 promoter activity was evaluated using chromatin immunoprecipitation (CHIP) and dual-luciferase reporter assay. We also treated NRK52E with ADAM10-specific over-expression vector and inhibitors and measured E-cadherin and α-SMA expression. In vivo, Wistar rats (*n* = 36) were subjected to unilateral ureteral obstruction (UUO) (*n* = 18) or sham surgery (*n* = 18), with tissues from post-operative day 3, 7, and 14 days examined, and PAX2/ADAM10 activity measured. ADAM10 expression was also assessed in kidneys from patients with chronic kidney disease (CKD).

**Results:**

In NRK52E overexpressing PAX2, ADAM10 and α-SMA levels were increased, while E-cadherin levels were decreased. CHIP and dual-luciferase reporter assay showed that PAX2 directly bound to a specific site within the ADAM10 promoter, and over-expression of PAX2 significantly activated ADAM10 transcription. NRK52E with ADAM10 over-expression also significantly decreased E-cadherin and increased α-SMA levels. In the fibrotic kidneys of rats with UUO, E-cadherin levels were increased and α-SMA levels were decreased, and expression of PAX2 and ADAM10 increased. ADAM10 expression also elevated in the renal tissues of CKD patients.

**Conclusions:**

PAX2 directly increased expression of ADAM10, the presence of which contributed to EMT in renal tubular epithelia and hence plays an important role in renal fibrosis.

## Introduction

Chronic kidney disease (CKD), characterized by renal fibrosis, is increasingly recognized as a global health issue due to its high prevalence and the devastating sequelae of end-stage renal disease, accompanied by a higher risk of cardiovascular events and premature death [1]. Renal fibrosis is associated with cortical parenchymal loss from epithelial degeneration, the accumulation of myofibroblasts and inflammatory cells, and the deposition of collagen fibers. Epithelial-to-mesenchymal transition (EMT), an important process related to renal fibrosis [2], refers to the pathogenic changes in epithelial lineage cells, including loss of structural polarity and the basement membrane contact, followed by morphological alterations producing spindle-shaped cells, similar to mesenchymal/myofibroblast cells [3]. EMT involves four pivotal stems: (1) loss of epithelial adhesion markers, such as the E-cadherin adhesion complex, (2) de novo expression of α-smooth muscle cell actin (SMA) and actin re-organization, (3) matrix metalloproteinases-induced basement membrane disruption, and (4) increased cell migration and invasion into the nearby interstitium [4]. Polarity loss during the early stages of EMT disrupts inter-cellular contacts [5]. The activation of mesenchymal markers is accompanied by epithelial marker suppression [5]. This is followed by actin architecture re-organization, loss of cellular cobblestone morphology, and adoption an elongated, spindle shape. Cellular morphological changes occur concomitantly with α-SMA expression [5]. Although the association between EMT and fibrosis has been firmly established [4], the underlying molecular mechanisms remain largely unknown.

The transcription factor, paired box 2 (PAX2), plays a key role in regulating kidney development. PAX2 expression is high during nephrogenesis, and its expression is required for tubular differentiation and branching [6]; however, PAX2 is gradually silenced in mature glomerular epithelia, and distal, and proximal tubules. Existing literature suggests that PAX2 may act as a transcriptional regulator due to its nuclear localization and its ability to bind DNA in vitro [7]. We previously observed that PAX2 was reactivated in renal tubular epithelial cells in a rat model of unilateral ureteral obstruction (UUO) [8]; moreover, PAX2 could induce EMT in vitro in tubular epithelia [9]. These results arouse the suspicion that PAX2 might contribute to renal interstitial fibrosis (RIF). The mechanisms by which PAX2 induces EMT are still not well understood, although some studies have suggested that PAX2 regulates ADAM10 expression in cancer cell lines including renal cell carcinoma [10] and melanoma [11]. As PAX2 was observed to induce EMT in renal tubular epithelial cells, and PAX2 could regulate ADAM10 in several cell types, we hypothesized that PAX2 may regulate ADAM10 activity in the renal tubular epithelia, and that ADAM10 might be implicated in the pathogenesis of EMT and renal fibrosis.

In the current study, we investigated the influence of ADAM10 on PAX2-induced EMT in renal tubular epithelia and the effect of ADAM10 in renal fibrosis. Our results, for the first time, indicate that PAX2 promotes EMT in the renal tubular epithelia via directly activating ADAM10, and that ADAM10 participates in the pathogenesis of renal fibrosis.

## Methods

### In vitro cellular culture

A rat renal tubular epithelial cell line (NRK52E) [8] was cultured at 37 °C in DMEM (GIBCO, Grand Island, NY, USA) with 10% fetal calf serum (Invitrogen, Carlsbad, CA, USA). We also cultured human proximal tubular epithelial cell line (HK-2) at 37 °C in DMEM with 10% fetal calf serum (Invitrogen) and 1% non-essential amino acids (NEAA; GIBCO).

### Plasmid construction and transfection of cells

Our target gene, *PAX2*, was designed and synthesized according to a GeneBank sequence (Gene ID: 293992). *Bam*HI and *Age*I were added to cut the 3′ and 5′ end of the *PAX2* gene, respectively. Both *PAX2* and lentiviral vector (pGC–LV) (Sangon Biotech, Shanghai, China) were digested by *Bam*HI and *Age*I, and DNA fragments were analyzed by gel electrophoresis. Digested products were purified and ligated with T4 DNA ligase, followed by co-transfection into *E. coli* DH5α cells (TIANGEN BIOTECH, Beijing, China). After amplification, the plasmid was extracted, cloned, and co-transfected with recombinant lentivirus plasmids, pHelper1.0 and pHelper2.0, into HEK 293T using Lipofectamine 2000 (Invitrogen). Supernatants were collected after 48 h and the titer of recombinant lentiviral vector (pGC–LV–PAX2) was determined. The vector pGC–LV–PAX2 was diluted in DMEM without serum, and added to NRK52E cells. Following incubation for 8–12 h, these cells were further cultured for 48 h in complete medium and observed under an inverted fluorescence microscope (TH4-200, Olympus, Japan).

The recombinant mammalian expression vector, pRK5M–ADAM10, was a kind gift from Rik Derynck [12] (Addgene plasmid #31717), and was transfected into HK-2 cells using Lipofectamine 3000 (Invitrogen).

### The preparation of protein and western blot

Harvested fresh tissue (50 mg) or cells (2 × 10^6^) were placed into 1.5 mL-tubes with 150 µL lysis buffer (50 mmol/L Tris–HCl, 150 mmol/L NaCl, 1% sodium deoxycholate, 0.1% sodium dodecylsulfate [SDS], 1% Triton X-100, and 1 mmol/L phenylmethylsulfonyl fluoride, in pH 7.4). Tissues were homogenized at 4 °C by ultrasonic vibration and centrifuged at 12,000 rpm for 30 min. We centrifuged cell lysates at 4 °C for 20 min at 12,000 rpm. The protein concentration of supernatants were measured by a Pierce™ BCA protein assay (ThermoFisher Scientific, Waltham, MA, USA).

Total proteins (40 µg) were electrophoresed on 10% SDS–polyacrylamide gels, transferred to polyvinylidene fluoride membranes (Millipore, Billerica, MA, USA), which were blocked in Tris-buffered saline (20 mmol/L Tris, 0.15 mol/L NaCl, 0.1% Tween 20, in pH 7.0) with 10% fat-free milk. Membranes were treated with primary antibody, including anti-ADAM10 (1:1000; Abcam, Cambridge, MA, USA; ab124695), anti-PAX2 (1:200; Santa Cruz Biotechnology, Dallas, TX, USA; sc-130387), anti-E-cadherin (1:1000; Abcam; ab76055), or anti-α-SMA (1:1000; Abcam; ab5694). After washing, we incubated membranes with horseradish peroxidase-conjugated anti-mouse or anti-rabbit secondary antibody (Santa Cruz Biotechnology, 1:2000 dilution). We visualized protein bands using Pierce™ ECL western blotting substrate (ThermoFisher Scientific), an enhanced chemiluminescence system, and images were obtained using the FluorChem FC2 imaging system (Alpha Innotech, San Leandro, CA, USA). We performed western blotting for β-tubulin as an internal sample using rabbit polyclonal antibody (1:2000; Proteintech, Rosemont, IL, USA).

### Quantitative real time-polymerase chain reaction (PCR)

We extracted total RNA from cells (2 × 10^6^) by RNAiso Plus (TaKaRa, Shiga, Japan) based on the manufacturer’s protocol. We reverse transcribed total RNA (1 µg) into cDNA using a PrimeScript®RT reagent Kit (Takara). The specifically designed primers and amplicons used are shown in Table 1. We performed real-time PCR using a SYBR®Premix Ex Taq™ kit (Tli RNaseH Plus; Takara) based on the manufacturer’s protocol. We carried out reactions on a RT-PCR system (ABI PRISM 7500, Applied Biosystems, Foster City, CA, USA), and the relative expression of target genes was normalized to β-actin or glyceraldehyde-3-phosphate dehydrogenase, by the ${2^ - }^{{\Delta \Delta {C_{\text{t}}}}}$ method.

**Table 1:** Primers and amplicons used during real-time PCR

Primers	Sequences	Length of products (bp)
ADAM10 (rat)		
Forward	5′-GGTTTCATCCAGACTCGGGGT-3′	80
Reverse	5′-TGAAACGGCAGGATTCGGTCT-3′	
PAX2 (rat)		
Forward	5′-ACGAGACTGGCAGCATCAA-3′	104
Reverse	5′-CGGGTTCTGTCGCTTGTATT-3′	
E-cadherin (rat)		
Forward	5′-TGCTCCTACTGTTTCTACG-3′	111
Reverse	5′-CTTCTCCACCTCCCTCTT-3′	
α-SMA (rat)		
Forward	5′-AGCCAGTCGCCATCAGGAAC-3′	90
Reverse	5′-CCGGAGCCATTGTCACACAC-3′	
GAPDH (rat)		
Forward	5′-TTCAACGGCACAGTCAAGG-3′	114
Reverse	5′-CTCAGCACCAGCATCACC-3′	
ADAM10 (human)		
Forward	5′-ACCGAACTCTGCCATTTCACTC-3′	96
Reverse	5′-CTGAATGATCTGCACAGCCCC-3′	
E-cadherin (human)		
Forward	5′-TCGCTTACACCATCCTCAGCC-3′	149
Reverse	5′-GTCAGCAGCTTGAACCACCAG-3′	
α-SMA (human)		
Forward	5′-CCTGAAGAGCATCCCACCCTG-3′	143
Reverse	5′-AGGCATAGAGAGACAGCACCG-3′	
β-Actin (human)		
Forward	5′-AACACCCCAGCCATGTACGTT-3′	90
Reverse	5′-GTCACCGGAGTCCATCACGAT-3′	

### Bioinformatic analysis of PAX2 binding sites

We determined the genomic region, 2000 base pairs upstream of the *ADAM10* transcriptional start site, through a NCBI Genomic BLAST program, cut and pasted into the JASPAR (http://jaspar.genereg.net/) and the PROMO (http://alggen.lsi.upc.es/cgi-bin/promo_v3/promo/promoinit.cgi?dirDB=TF_8.3) software to locate putative PAX2 binding sites.

### Quantitative chromatin immunoprecipitation assay

A chromatin immunoprecipitation (CHIP) kit (EZ-ChIP™; #17–371; EMD Millipore) was used for a quantitative chromatin immunoprecipitation (qChIP) assay, based on the manufacturer’s manual. In brief, we fixed NRK52E transfected with pGC–LV–PAX2 using 1% paraformaldehyde, and then sheared chromatin derived from isolated nuclei. Each incubation mixture contained 100 µL of sheared chromatin, 60 µL protein G agarose beads, and 10 µg PAX2 antibodies (Abnova, Taipei, Taiwan). We used the following primer pairs, 5′-CATTGGGTACGGATGCGTCAC-3′ and 5′-GCCTC TTCCTTCCTCCCTTCC-3′, to amplify a 119-bp product of the human *ADAM10* promoter containing the putative PAX2 binding site (for sequence, referred to NM_001110.3).

### Dual-luciferase reporter gene assay

The ADAM10 promoter was synthesized and a luciferase reporter plasmid pGL3–ADAM10-luc was created based on the putative PAX2 binding site with its flanking 100- to 200-bp segments (sequences as follows: 5′-GCGGGGCGGGAGGCAGGGGCGCGGCCTCGCGAGTGCATTGGGTACGGATGCGTCACGTGGCGAGAAAGGAGGCGGAGGGCCGCGGCTCAGGGAAGGGGCTGAGACCAGGCGAAGAGCGAGGGCCGGGAAGCGGGGAAGGGAGGAAGGAAGAGGC-3′). We also added the *Kpn*I and *Hin*dIII restriction sites upstream and downstream of the synthetic segment (Biotechnology Inc., Shanghai). Vector construction was verified by 1% SDS gel electrophoresis. The gel band was excised, mixed with T4 ligase at 16 °C overnight, and used to transfect susceptible DH5α cells. Clones were selected, and fragment sequences were confirmed via *Kpn*I and *Hin*dIII cutting, yielding the pGL3–ADAM10-luc plasmids.

We further transfected pGC–LV–PAX2-carrying NRK52E in a 96-well plate (10,000 cells/well) with pGL3–ADAM10-luc (100 ng) using Lipofectamine 3000 (Invitrogen), with pRL–TK (10 ng) (Promega) as a control, in order to determine transfection efficiency in serum-free DMEM. We measured luciferase activity 48 h after transfection by the Dual Luciferase Report Assay System (Promega). Firefly luciferase activity was normalized to Renilla luciferase activity in each well. All experiments were performed in triplicate.

### Immunofluorescence staining and immunohistochemistry

Cells cultured on coverslips were washed three times at room temperature with cold phosphate-buffered saline (PBS) and fixed with cold 4% paraformaldehyde for 10 min. After washing three times with PBS containing 0.1% Triton X-100, we blocked cells using goat serum for 1 h at room temperature and incubated them with primary antibodies against ADAM10 (1:100; OriGene Technologies, Rockville, MD, USA), E-cadherin (1:100; Abcam; ab76055), or α-SMA (1:100; Abcam; ab5694). Goat anti-rabbit IgG (H+L; 1:2000; Alexa Fluor 594; Invitrogen) and goat anti-mouse IgG (H+L; 1:2000; Alexa Fluor 488; Invitrogen) were used as secondary antibodies. We used non-immune IgG as the negative control. Stained cells were mounted with antifade mounting medium (Beyotime Institute of Technology, Haimen, China) and visualized with an Olympus BX-60 epifluorescence microscope equipped with a digital camera.

We dewaxed, rehydrated, and incubated tissue slides (4–5 µm thick) with 3% hydrogen peroxide for 10 min, and blocked them in 10% normal rabbit or goat serum for 1 h for immunohistochemical examination. For immunofluorescence staining, 3% hydrogen peroxide was omitted. We then incubated slides at 4 °C overnight with primary antibodies, against ADAM10 (1:50; OriGene), E-cadherin (1:50; Abcam; ab76055), α-SMA (1:100; Abcam; ab5694), or PAX2 (1:50; Santa Cruz Biotechnology; sc-130387) for immunohistochemical examination, or ADAM10 (1:100; Diaclone, Besancon, France) for immunofluorescence staining. Sections were incubated with biotinylated goat anti-rabbit or anti-mouse IgG antibody as the secondary antibody for immunohistochemical examination, and goat anti-mouse IgG (H+L; 1:2000; Alexa Fluor 488; Invitrogen) for immunofluorescence staining. In this experiment, we also used non-immune IgG as a negative control. Slides were counterstained with hematoxylin, and then dehydrated and mounted for immunohistochemical examination.

### UUO animal model

Male Wistar rats (*n* = 36, six per group) weighing 120–150 g were obtained from the Department of Experimental Research Animal Laboratory, Shengjing Hospital of China Medical University (Shenyang, China). We performed UUO as described previously [8]. Rats were sacrificed at day 3, 7, and 14 after surgery, and their kidneys were harvested. Animals were handled in accordance with guidelines for the care and use of experimental animals established by the Ethics Committee of Shengjing Hospital.

### Renal biopsies

We obtained renal biopsy samples from patients diagnosed with CKD between 2011 and 2014 at the Pediatric Nephrology Department of Shengjing Hospital of China Medical University. We identified formalin-fixed and paraffin-embedded samples showing chronic interstitial nephritis (*n* = 2) or focal and segmental glomerulosclerosis (*n* = 3) from the Pathology Department of Shengjing Hospital. We used samples from living kidney donor biopsies as controls. The study was approved by the Ethics Committee of Shengjing Hospital.

### Statistical analysis

All statistical analyses were performed using SPSS 18.0 software. An independent samples *t* test was used to test for statistical significance, and *P* < 0.05 was considered to indicate statistical significance.

## Results

### Synthesis of the PAX2 genetic segment and its vector

**Fig. 1:**
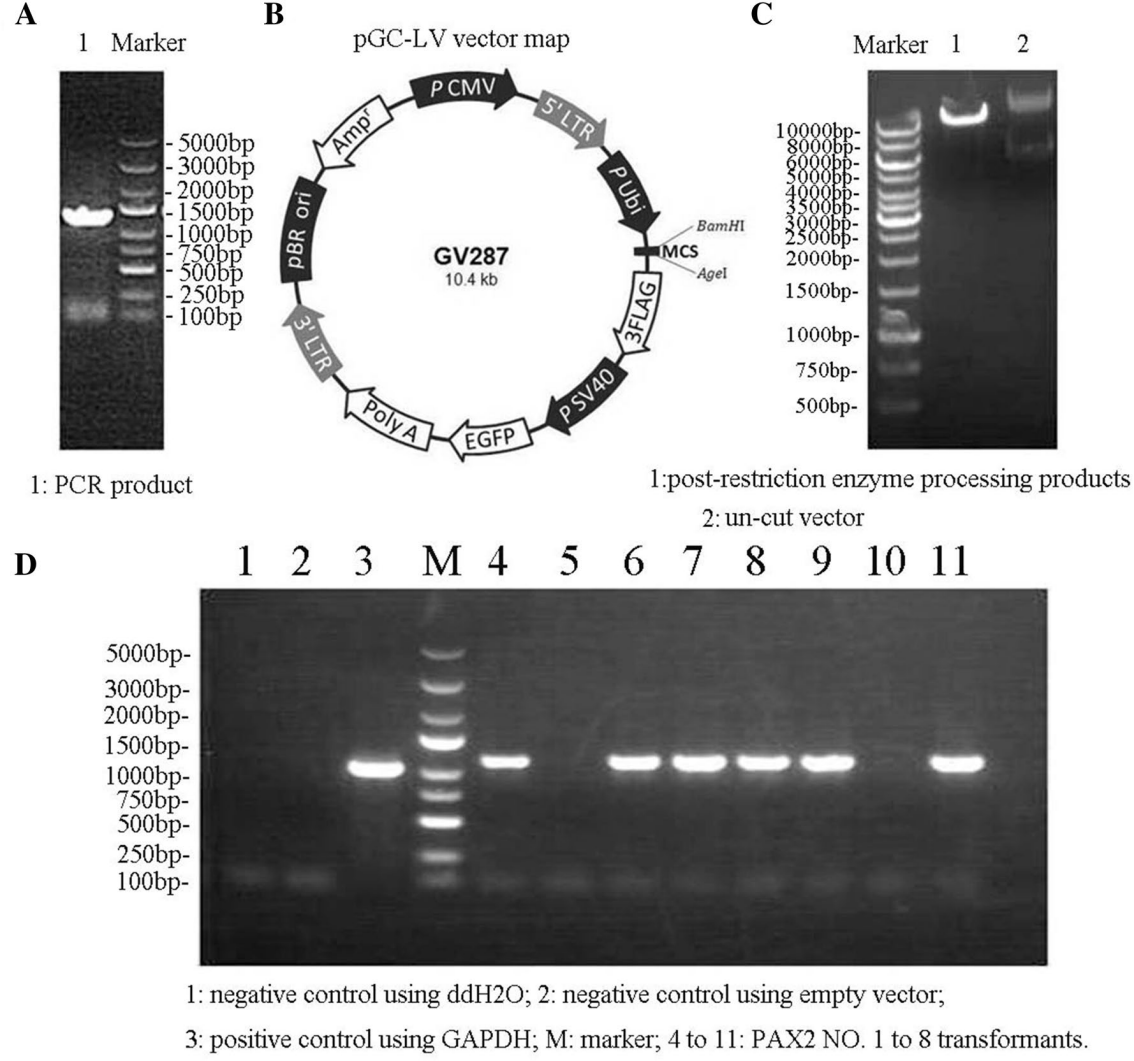
Synthesis of the PAX2 gene and construction of a lentivirus-based expression vector. **a** Electrophoresis results of PAX2 gene synthesis. 1, PCR product; M, DNA ladder (bands from upper to lower: 5000, 3000, 2000, 1500, 1000, 750, 500, 250, and 100 bp). **b** pGC–LV vector map. **c** Electrophoresis results after restriction enzyme digestion. M, 10 kb marker (bands from upper to lower: 10, 8, 6, 5, 4, 3.5, 3, 2.5, 2, 1.5, 1 kb, 750, 500 bp). 1, post-restriction enzyme processing products; 2, un-cut vector. **d** Bacterial colony electrophoresis results. 1, negative control using ddH_2_O; 2, negative control using empty vector; 3, positive control using GAPDH; M, marker (bands from upper to lower, 5, 3, 2, 1.5, 1 kb, 750, 500, 250, 100 bp). 4–11, PAX2 NO. 1–8 transformants

The whole sequence of rat *PAX2* was obtained from the NCBI database (Gene ID: 293992and designed specific primers based on its full-length coding sequence (CDS) (Fig. 1a) including *Bam*HI and *Age*I restriction sites. The 1292-bp *PAX2* gene was cloned into the pGC–LV vector (Ubi-MCS-3FLAG-SV40-EGFP, with *Bam*HI and *Age*I) (Fig. 1b, c). Transfected clones were verified using PCR, and exchanged the products into linear expression vectors, with which susceptible cells were transfected and colonies were validated (Fig. 1d). The sequence of the synthetic PAX2 genetic segments and that of the open reading frame were reassured by PCR on the isolated clones, followed by lentivirus package and titer determination of the pGC–LV–PAX2 plasmid.

### PAX2 over-expression

**Fig. 2:**
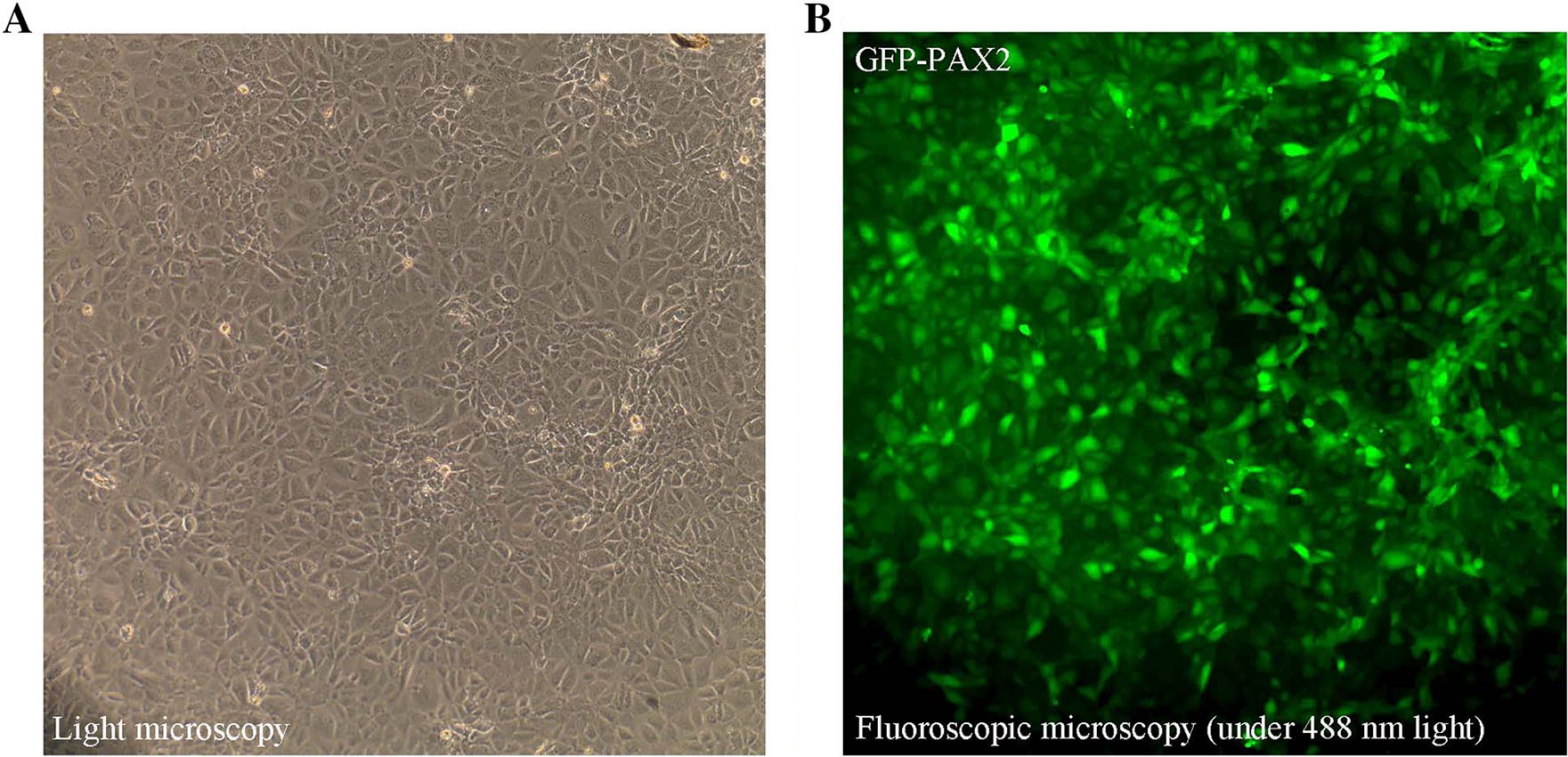
Expression of pGC–LV–PAX2 eukaryotic vectors in rat renal tubular epithelia (100 ×), with a transfection efficiency > 80%. **a** Light microscopy, **b** fluoroscopic microscopy (under 488 nm light)

**Fig. 3:**
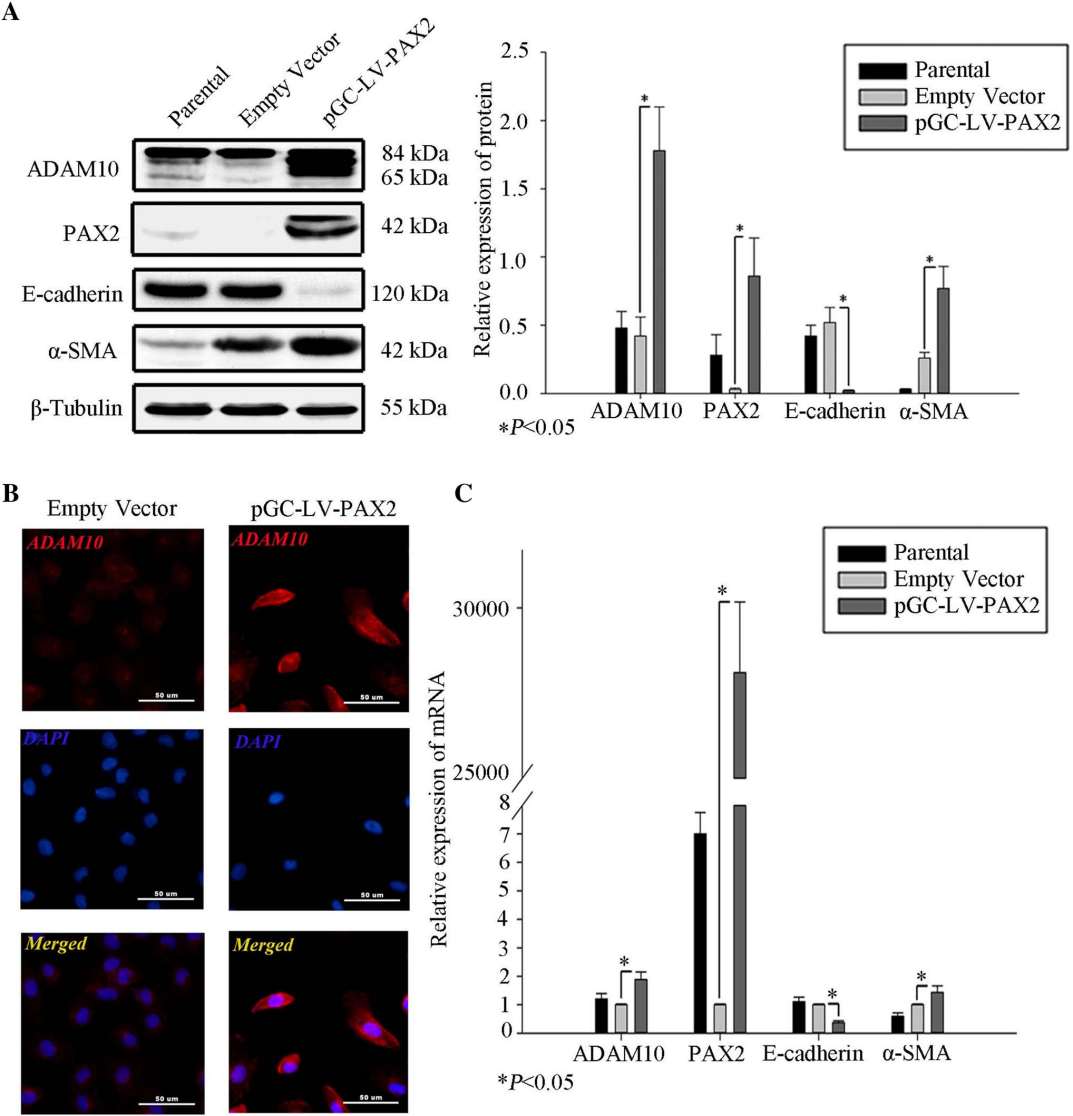
PAX2 influences ADAM10 expression. **a** Total cell lysates of NRK52E transfected with pGC–LV–PAX2, assessed by western blot. Protein levels were normalized to β-tubulin. Data represent mean ± SD (*n* = 3). **b** ADAM10 expression (red) analyzed by immunofluorescence in NRK52E transfected with pGC–LV–PAX2 or empty vectors. The nuclei of NRK52E cells are stained with 4′,6-diamidino-2-phenylindole (DAPI). Scale bars represent 50 µm. **c** Messenger RNA isolated from NRK52E transfected with pGC–LV–PAX2 or empty vectors. Data represent mean ± SD (*n* = 3). *P < 0.05 compared to vector control

NRK52E cells were transfected with pGC–LV–PAX2 and intracellular PAX2 expression was observed via fluorescent microscope. The intensity of green fluorescence was highest 72 h after transfection, and the transfection efficiency exceeded 80% (Fig. 2a, b). Real-time PCR and western blot (Fig. 3a, c) revealed similar results. These findings suggest that pGC–LV–PAX2 induced over-expression of PAX2 in rat renal tubular epithelia, and the efficiency peaked 72 h post transfection.

### PAX2 may influence ADAM10 expression in tubular epithelial cells

To investigate whether PAX2 could influence ADAM10 expression in tubular epithelial cells, over-expressed PAX2 in NRK52E by transfection with pGC–LV–PAX2. Up-regulating PAX2 led to a markedly increased expression of ADAM10 (Fig. 3a), while PAX2 was found to directly regulate ADAM10 using immunofluorescence and quantitative RT-PCR (Fig. 3b, c, respectively). Up-regulating PAX2 led to a reduction of E-cadherin expression and an increase in α-SMA expression (Fig. 3a, c), similar to our previous observations [9].

### PAX2 binds to the ADAM10 promoter

**Fig. 4:**
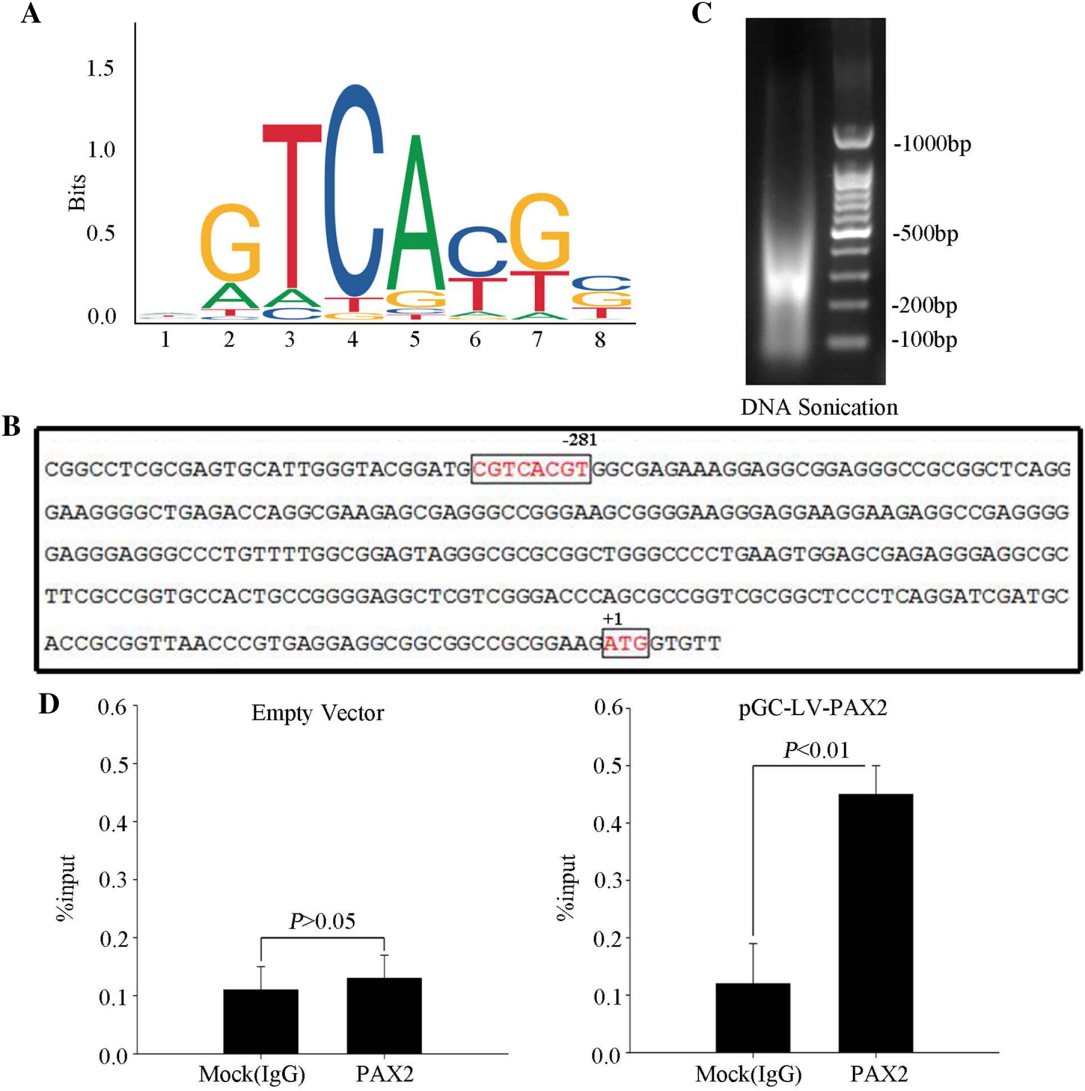
PAX2 directly binds the *ADAM10* promoter. **a** A PAX2 consensus binding motif from the JASPAR CORE database (http://jaspar.genereg.net/). **b** The putative PAX2 binding site (− 281 bp) within the *ADAM10* promoter of rat renal tubular epithelial cells. **c** DNA sonication revealed that the majority of DNA had been sheared to fragments between 200 and 500 bp in length. **d** Results of a qChIP assay of the *ADAM10* promoter region in NRK52E cells transfected with pGC–LV–PAX2 compared to those transfected with empty vectors. Data represent mean ± SD (*n* = 3). IgG was used as a control

To identify whether PAX2 regulates ADAM10 in renal tubular epithelial cells, we computationally analyzed the *ADAM10* promoter region. We identified a putative PAX2 binding site (− 281 bp) (Fig. 4a, b) and performed the qChIP assay (Fig. 4d) to confirm whether PAX2 directly binds *ADAM10*. We also amplified the *ADAM10* promoter carrying the putative PAX2 binding site in NRK52E transfected with pGC–LV–PAX2, in which proteins were subsequently immunoprecipitated with PAX2 antibodies, but not with control IgG (Fig. 4d).

### PAX2 increases ADAM10 promoter activity in renal tubular epithelial cells

**Fig. 5:**
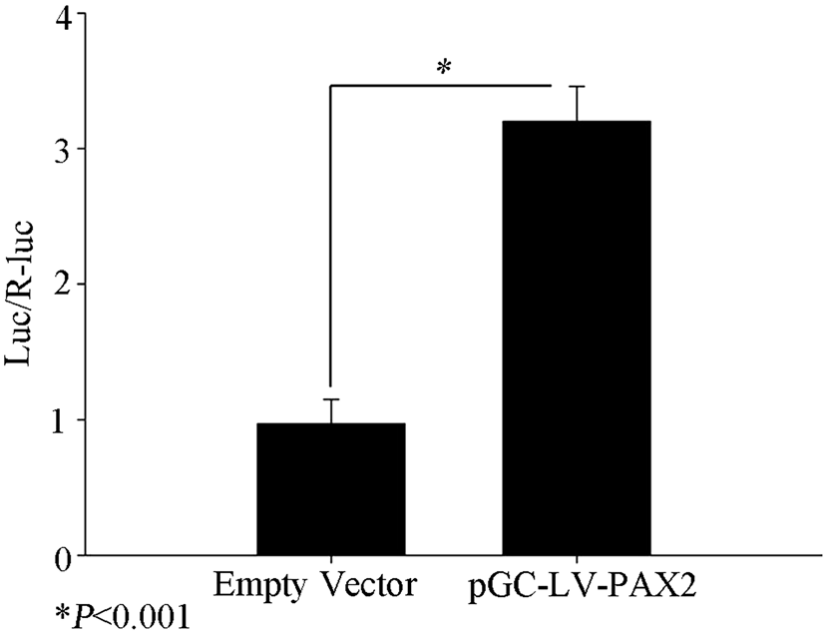
Comparison of luciferase activity in renal tubular epithelial cells with and without PAX2 over-expression (*P < 0.001)

To evaluate the effect of PAX2 binding to the ADAM10 promoter, we used a dual-luciferase reporter assay. NRK52E cells were transfected with pGC–LV–PAX2 or co-transfected with pGL3–ADAM10-luc and pRL–TK control vectors. Luciferase activity was assessed 48 h after transfection. We found ADAM10 levels to be significantly higher in PAX2-overexpressing cells than controls (*P* < 0.001; Fig. 5), suggesting that PAX2 regulates ADAM10 expression in rat renal epithelial cells.

### ADAM10 induces EMT in tubular epithelia

**Fig. 6:**
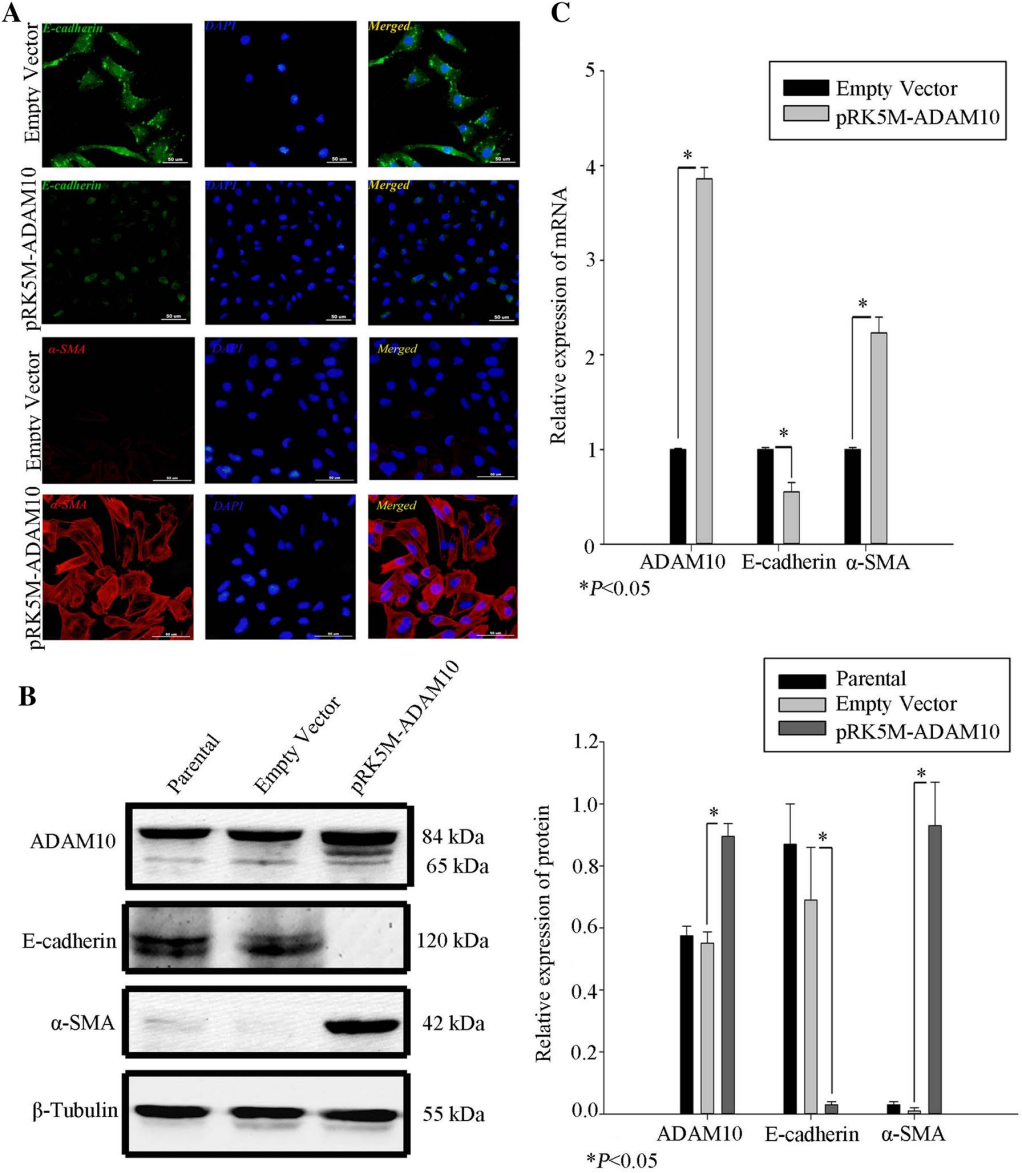
ADAM10 induces EMT. **a** E-cadherin (green) and α-SMA expression (red) were analyzed by immunofluorescence in HK-2 transfected with pRK5M–ADAM10 or empty vectors. HK-2 nuclei were stained with DAPI. Scale bars represent 50 µm. **b** Total cell lysates of HK-2 transfected with pRK5M–ADAM10 or empty vectors were prepared. Protein expression was normalized to β-tubulin. Data represent mean ± SD (*n* = 3). **c** Messenger RNA was isolated from HK-2 transfected with pRK5M–ADAM10 or empty vectors. Data represent mean ± SD (*n* = 3). *P < 0.05 compared to vector control

To assess the function of ADAM10, we examined the impact of over-expressed ADAM10 in human proximal tubular epithelia. HK-2 was transfected with either an expression vector containing *ADAM10* or an empty vector, and E-cadherin and α-SMA expression was assessed via immunofluorescence (Fig. 6a). Over-expression of ADAM10 reduced staining of E-cadherin in the plasma membrane, whereas α-SMA expression staining dramatically increased in the cytoplasm. EMT was confirmed in ADAM10-overexpressing tubular epithelial cells by western blot and quantitative RT-PCR (Fig. 6b, c).

### ADAM10 partially contributes to PAX2-induced EMT in rat renal epithelia

**Fig. 7:**
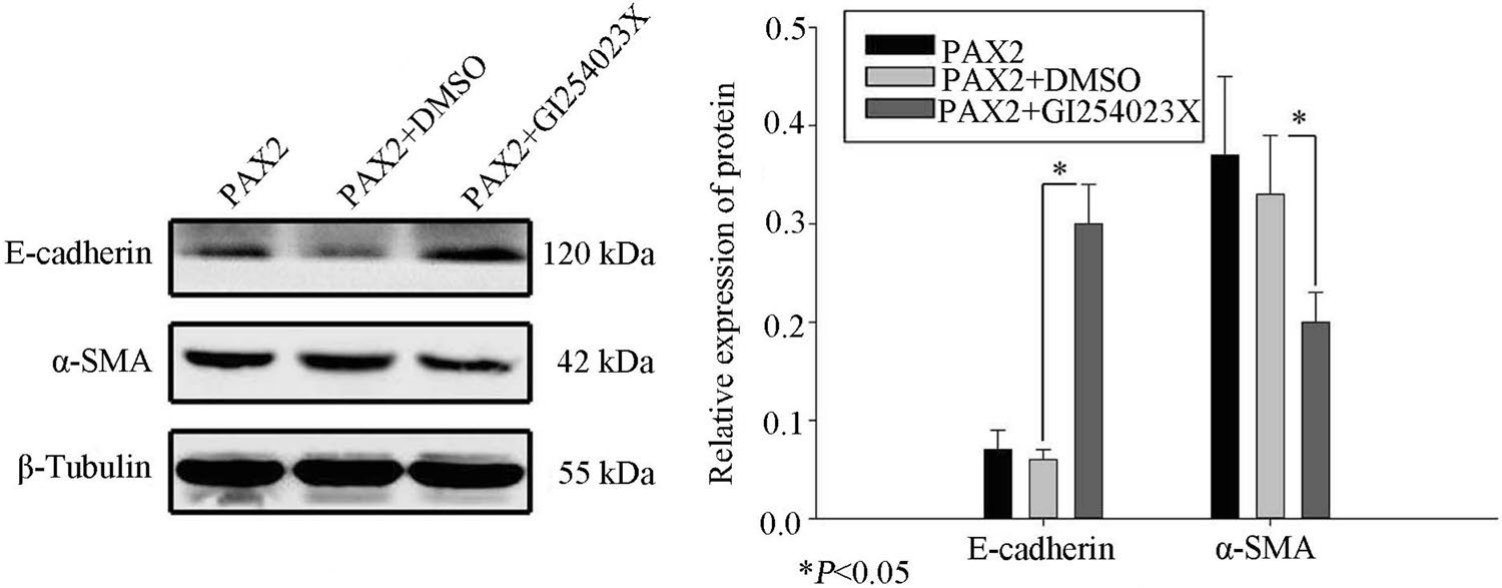
Western blot indicating E-cadherin and α-SMA levels in PAX2-overexpressing NRK52E treated with the ADAM10 inhibitor, GI254023X (*P < 0.05)

We added the ADAM10 inhibitor, GI254023X (Sigma-Aldrich, US), to PAX2-overexpressing NRK52E, and evaluated expression of E-cadherin and α-SMA by western blot. In GI254023X-treated cells, expression of E-cadherin was significantly higher and expression of α-SMA was lower than in vehicle (DMSO)-treated cells (*P* < 0.05; Fig. 7), without complete reversal of PAX2-induced EMT.

### ADAM10 contributes to UUO-induced renal fibrosis

**Fig. 8:**
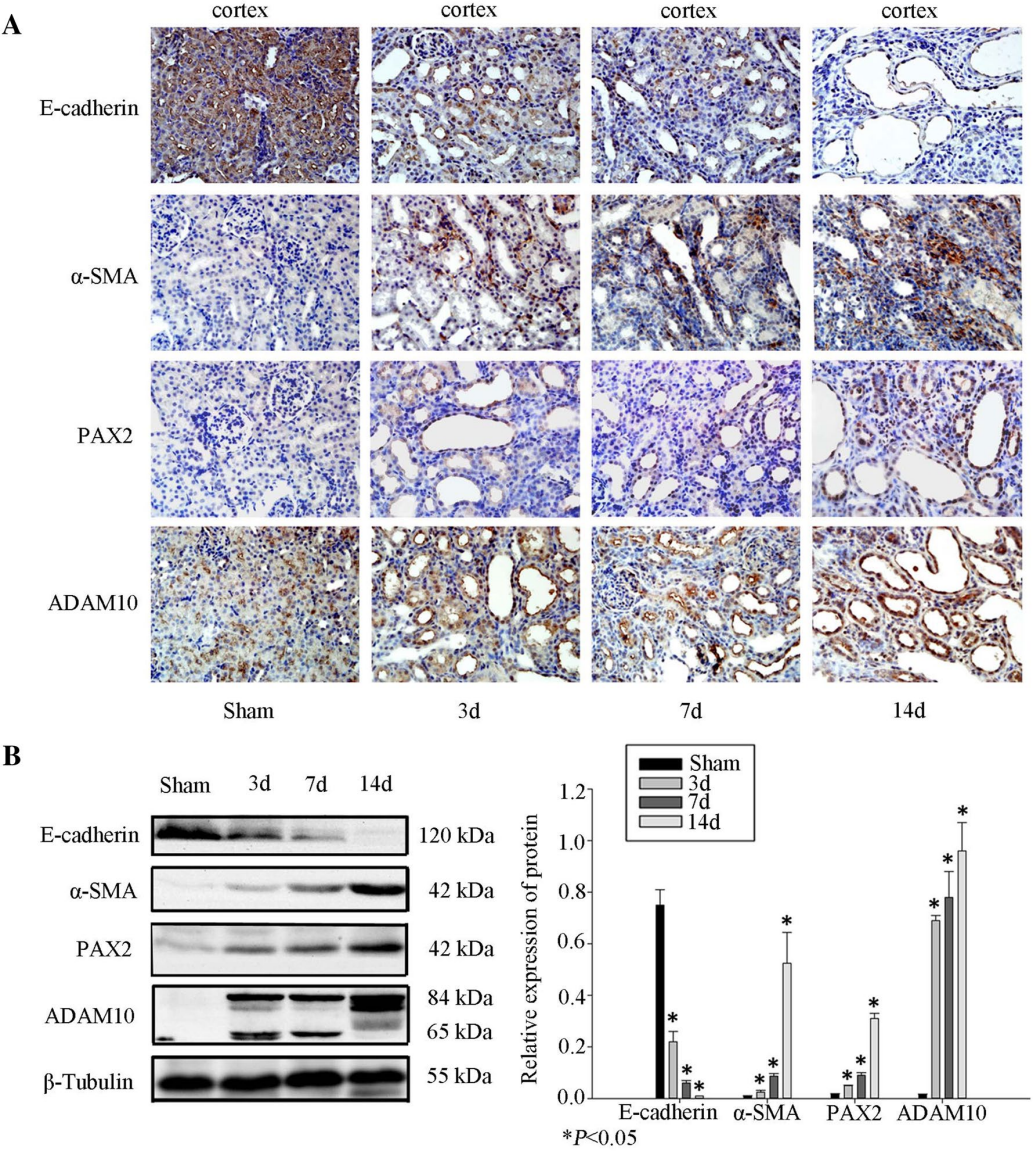
ADAM10 participates in UUO-induced renal fibrosis. **a** Immunohistochemical staining of E-cadherin, α-SMA, PAX2, and ADAM10 in the renal tissues of sham surgery and UUO rats. Original magnification, × 400. **b** Total kidney lysates from rats that underwent UUO or sham surgery rats were prepared. Protein expression was assessed by western blot relative to β-tubulin. Data represent mean ± SD (*n* = 3). *P < 0.05 compared to the sham group

To investigate the role of ADAM10 in renal fibrosis in vivo, we assessed the renal expression of ADAM10 in a rat model of UUO. Immunohistochemical staining and western blot revealed that E-cadherin was decreased and α-SMA expression was increased in the kidneys of UUO rats in a time-dependent manner. This observation suggests that renal tubular epithelia underwent EMT during renal fibrosis (Fig. 8a, b). ADAM10 was predominantly located in the plasma membrane of renal tubular epithelia, and was significantly induced in the fibrotic kidney in a time-dependent manner (Fig. 4a), consistent with PAX2 expression. Western blot also revealed that renal ADAM10 was significantly induced 3 days after UUO, consistent with PAX2 expression (Fig. 8b) and, at times, preceded the onset of EMT [13].

### ADAM10 is over-expressed in the renal tissues of CKD patients

**Fig. 9:**
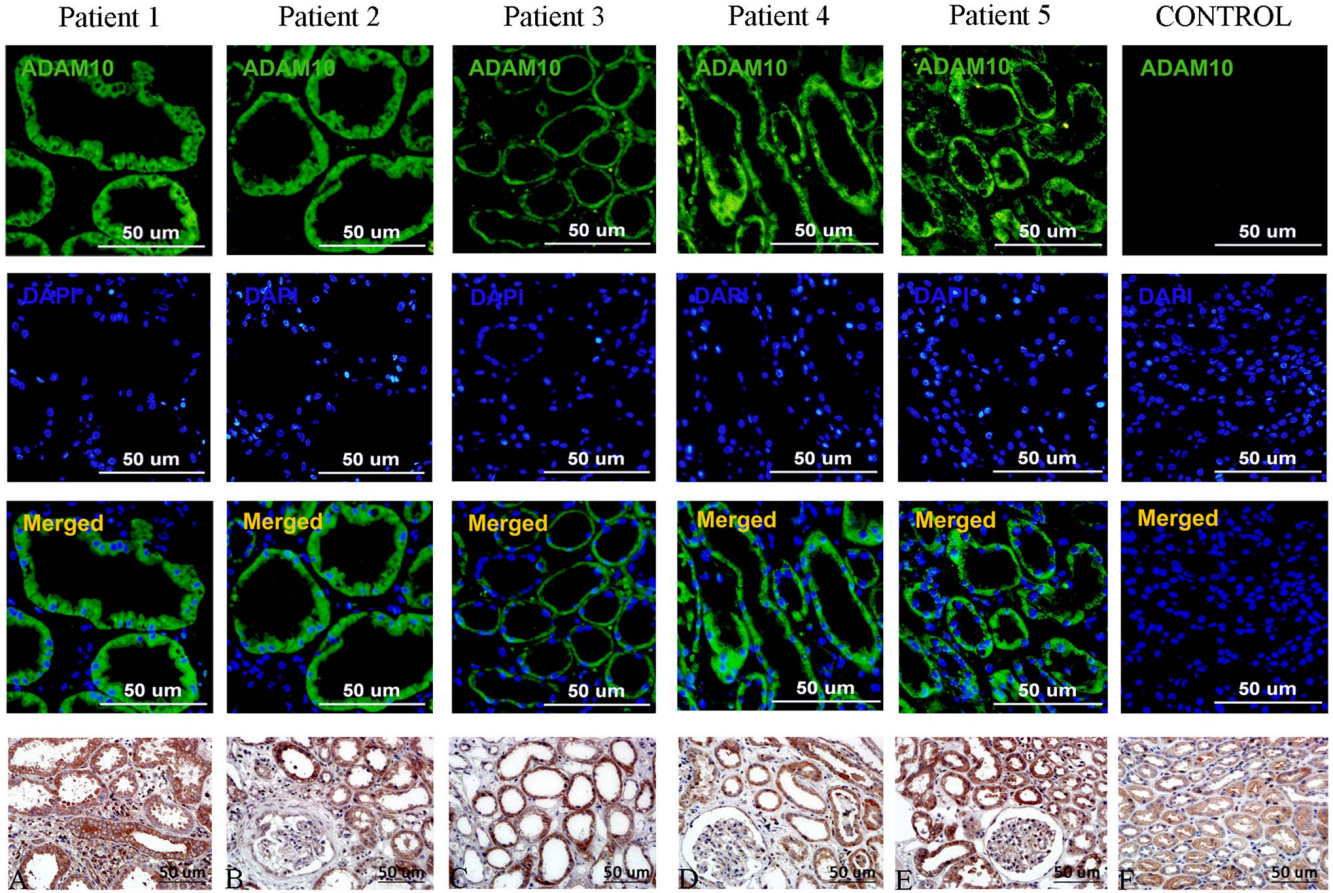
ADAM10 expression in human renal tissues. ADAM10 expression (green) in renal biopsy samples from CKD patients (stages 2–3) was analyzed by immunofluorescence, with controls derived from living donor biopsies. Cell nuclei were stained with DAPI. Scale bar represents 50 µm. **a**–**e** Immunohistochemical staining of ADAM10 in the renal tissues of renal biopsy samples from CKD patients (stages 2–3). Original magnification, × 400

As we observed ADAM10 to be activated in a rat renal fibrosis model, we next assessed ADAM10 expression in renal biopsy tissues from five CKD patients (stages 2–3) with tubulointerstitial injury and 1 without CKD. ADAM10 expression was assessed in paraffin-embedded renal tissues from 2 patients with chronic interstitial nephritis, 3 with focal segmental glomerulosclerosis, and 1 living donor renal biopsy. ADAM10 expression was higher in the diseased kidneys from CKD patients than normal controls, indicating that ADAM10 correlates with tubulointerstitial fibrosis in CKD patients (Fig. 9).

## Discussion

In this study, we demonstrated that ADAM10 plays an important role in PAX2-induced EMT in the renal tubular epithelia. We showed that ADAM10 expression was up-regulated during PAX2-induced EMT. Moreover, PAX2 directly bound the *ADAM10* promoter region and increased its transcriptional activity. In addition, over-expression of ADAM10 could induce EMT in renal tubular epithelia, and partially contributed to PAX2-induced EMT. Finally, ADAM10 was up-regulated in the obstructive kidneys in a time-dependent manner and correlated with renal fibrosis in vivo. These observations, together with our previous findings [9], suggest that PAX2–ADAM10 signaling contributes significantly to the pathogenesis of RIF.

PAX2, a nuclear transcription factor, has recently been found to be expressed in the renal interstitium and participates in the development and progression of RIF [14]. Timely expression of PAX2 is required for normal kidney development [15]. Several groups have found that PAX2 expression closely correlates with various kidney diseases. Mure et al. [16] found that PAX2 was highly expressed in the nephrogenic zone, but its expression was found to decrease progressively in the control kidneys of a fetal lamb UUO model. Huang et al. [17] studied the potential role of PAX2 in EMT within the remnant kidneys of 5/6 nephrectomized rats and in cultured renal tubular epithelia. They discovered that re-expression of PAX2 in renal tubular epithelial cells plays an important role in the pathogenesis of EMT. These results conform with our previous findings [8, 9]. However, the molecular mechanisms by which PAX2 induces EMT in renal tubular epithelia remain poorly understood.

ADAM10 is regulated by transcription factor PAX2 in renal cell carcinoma [10] and melanoma cells [11]. We further demonstrated that PAX2 could activate ADAM10 transcription in renal tubular epithelial cells. Our conclusions are based on the following observations: first, ADAM10 was up-regulated in NRK52E after transfection with PAX2. Second, computational analysis showed that the *ADAM10* promoter contains a PAX2 binding site, which was confirmed by the ChIP assay and dual-luciferase reporter gene assay. Third, ADAM10 was over-expressed in the fibrotic kidneys of patients with CKD and in a UUO model in vivo. In addition, ADAM10 was expressed in a time-dependent manner, consistent with PAX2 expression in the UUO model.

ADAM10 is a zinc-dependent transmembrane protease that regulates Notch, EGF, E-cadherin, and other signaling pathways. Maretzky et al. [18] have reported that over-expression of ADAM10 in renal tubular epithelial cells increased the cleavage of E-cadherin, reducing cell–cell adhesion and increased epithelial cell migration. The ADAM10/E-cadherin interaction serves to regulate inflammatory epidermal diseases, and is characterized by E-cadherin loss, followed by epithelial integrity impairment [19]. Park et al. [20] suggested that ADAM10 induced EMT in Epstein–Barr virus-infected retinal pigment epithelial cells. In contrast, down-regulation of ADAM10 produces a flaccid morphological appearance, up-regulation of Slug and E-cadherin loss, as observed during EMT in the renal cell carcinoma cells [10]. However, the regulation and function of ADAM10 in renal fibrosis still remains unclear.

This study provides evidence that ADAM10 is involved in the EMT of renal tubular epithelia and renal fibrosis. Over-expression of exogenous ADAM10 leads to E-cadherin loss and increases α-SMA in HK-2 cells, while inhibiting ADAM10 can partially reverse PAX-2-induced EMT. Immunochemical staining indicated that PAX2 expression was increased in the renal tubular epithelia on days 3, 7, and 14 after ureteral ligation, consistent with previous work [8]. Additionally, western blot also indicated that ADAM10 protein levels were up-regulated in these cells 3, 7 and 14 days after ligation. Indeed, western blotting of fibrotic kidneys induced by UUO revealed a marked time-dependent induction of ADAM10. Moreover, we revealed that ADAM10 expression was increased in the renal tubulointerstitial parts of patients with chronic interstitial nephritis and focal segmental glomerulosclerosis. In this sense, our data provide the first clear in vitro and in vivo evidence that ADAM10 is involved in EMT of renal tubular epithelial cells and in renal fibrosis. Another important substrate of ADAM10 is the Notch receptor and ligand, Delta [21], which also plays a crucial role in renal fibrosis. Djudjaj et al. [22] reported that activated Notch-3 receptor protein was detected in all obstructed kidneys on day 14 following UUO, whereas in the unobstructed kidneys no Notch-3 receptor protein was detected. Xiao et al. [23] found that the protein levels of intracellular domain of Notch 1–4, and its downstream effectors Hes1 and HeyL increased 7 days after UUO. However, whether ADAM10 activates the Notch signaling pathway in renal fibrosis requires further investigation.

In summary, our findings shed light on the mechanism of PAX2-induced EMT and renal fibrosis and the up-regulation of ADAM10. The contribution of ADAM10 over-expression to PAX2-induced EMT deepens our understanding of renal fibrosis and may inform development of anti-fibrogenic strategies.
